# Avian Influenza A(H5N1) Isolated from Dairy Farm Worker, Michigan, USA 

**DOI:** 10.3201/eid3106.250386

**Published:** 2025-06

**Authors:** Nicole Brock, Joanna A. Pulit-Penaloza, Jessica A. Belser, Claudia Pappas, Xiangjie Sun, Troy J. Kieran, Hui Zeng, Juan A. De La Cruz, Yasuko Hatta, Han Di, C. Todd Davis, Terrence M. Tumpey, Taronna R. Maines

**Affiliations:** Centers for Disease Control and Prevention, Atlanta, Georgia, USA

**Keywords:** Influenza, H5N1, highly pathogenic avian influenza, zoonoses, respiratory infections, ferret, clade 2.3.4.4b, Michigan, United States

## Abstract

Influenza A(H5N1) viruses have been detected in US dairy cow herds since 2024. We assessed the pathogenesis, transmission, and airborne release of A/Michigan/90/2024, an H5N1 isolate from a dairy farm worker in Michigan, in the ferret model. Results show this virus caused airborne transmission with moderate pathogenicity, including limited extrapulmonary spread, without lethality.

Highly pathogenic avian influenza A(H5N1) clade 2.3.4.4b viruses have displayed unprecedented global spread among wild birds leading to numerous spillover infections in mammalian species. Of note, outbreaks in dairy cattle and gallinaceous birds have resulted in human infections in the United States during 2024–2025 (*1*). Increased frequency of H5N1 viruses crossing species barriers has caused concern that the avian influenza viruses are adapting to mammals. A critical component of influenza pandemic preparedness is early identification of emerging novel influenza viruses that cause disease and transmit efficiently in humans. A clade 2.3.4.4b H5N1 virus, A/Michigan/90/2024 (MI90), genotype B3.13, was isolated from a conjunctival swab specimen collected from a human patient in Michigan with conjunctivitis after exposure to infected cattle (*2*,*3*). In this article, we report the pathogenesis, transmission, and airborne exhalation of MI90 virus in ferrets, the standard animal model for influenza virus risk assessments (*4*).

We inoculated 18 ferrets with MI90 virus as previously described (*5*,*6*). We euthanized 3 ferrets on 3 and 5 days postinoculation (dpi) to assess virus spread in tissues. We used 6 ferrets to assess transmission in a cohoused, direct contact setting as a direct contact transmission model and through the air in the absence of direct or indirect contact as a respiratory droplet transmission model. We paired each ferret with a naive contact, as previously described (*4*). We observed clinical manifestations daily and collected nasal wash (NW), conjunctival, and rectal swab samples every 2 days postinoculation or postcontact. We confirmed transmission by testing for seroconversion to homologous virus in the contact animals.

Although all MI90-infected ferrets survived the 21-day study, we noted moderate disease. In inoculated ferrets, the mean maximum weight loss was 9.8%, fever (1.8°C above baseline) and lethargy were transient, and nasal and ocular discharge and sneezing were evident at 4–9 dpi (Table). We detected virus 3 dpi primarily in respiratory tract tissues; titers were highest in ethmoid turbinate samples (7.4 log_10_ PFU/mL) and at low levels in brain and gastrointestinal tissues. We observed similar results in tissues collected 5 dpi.

**Table:** Clinical signs and virus titers in ferrets infected with avian influenza A(H5N1) isolated from dairy farm worker in Michigan, 2024*

Clinical signs and tissues sampled	Inoculated ferrets			Transmission models		
	Euthanized at 3 dpi	Euthanized at 5 dpi		Inoculated	DCT	RDT
Weight loss, %†	4.5 (3/3)	11.8 (3/3)		9.8 (12/12)	5.5 (6/6)	6.6 (3/6)
Fever, °C above baseline‡	0.9 (3/3)	1.3 (2/3)		1.8 (11/12)	1.8 (6/6)	2.0 (3/6)
Nasal wash	6.1 (3/3)	5.4 (3/3)		5.1 (1–5 d)	4.6 (5–7 d)	4.5 (9–11 d)
Conjunctival wash§	1.4 (3/3)	NT		3.2 (3 d)	ND	ND
Rectal swab¶	1.4 (3/3)	NT		2.6 (3–5 d)	1.0 (3 d)	1.4 (3 d)
Tissues						
Nasal turbinate	6.6 (3/3)	5.3 (3/3)		NT	NT	NT
Ethmoid turbinate	7.4 (3/3)	6.5 (3/3)		NT	NT	NT
Soft palate	3.5 (1/3)	NT		NT	NT	NT
Lung#	3.5 (2/3)	4.3 (3/3)		NT	NT	NT
Trachea#	5.9 (3/3)	5.8 (2/3)		NT	NT	NT
Intestine#	1.8 (1/3)	ND (0/3)		NT	NT	NT
Brain#	2.4 (3/3)	2.4 (2/3)		NT	NT	NT
Olfactory bulb	3.1 (2/3)	4.2 (3/3)		NT	NT	NT

*Values are log_10_ PFU/mL (no. ferrets affected/total no. in group) except as indicated. DCT, direct contact transmission model; ND, not detected; NT, not tested; RDT, respiratory droplet transmission model. †Mean maximum weight loss after inoculation with 10^6^ PFU A/Michigan/90/2024 A(H5N1) virus in a 1-mL volume. ‡Mean maximum rise in body temperature from baseline (37.4°C–39.0°C). §Conjunctival washes collected from 6 of 12 inoculated animals and 3 each of DCT and RDT contact ferrets in the transmission experiment; number of ferrets with detectable virus or day of mean peak shown parenthetically. ¶Virus in rectal swab samples detected in 8 of 12 inoculated and 1 each of DCT and RDT contact ferrets in the transmission experiment; number of ferrets with detectable virus or day of mean peak shown parenthetically. #Values are log_10_ PFU/g.

During the direct contact transmission experiment, inoculated ferrets shed virus in NW that peaked at 4.7–5.4 log_10_ PFU/mL at 1–5 dpi (Figure, panel A). Four of 6 cohoused contact animals had virus in NW (peak 2.5–4.9 log_10_ PFU/mL) at 5–7 days postcontact, whereas all 6 contact animals had viral RNA detected (3.6–7.7 log_10_ copies/mL) in NW (*7*) and seroconverted to MI90 virus, indicating that transmission was 100% (6/6 animals). In the respiratory droplet transmission experiment, NW collected from inoculated animals peaked 2.6–4.8 log_10_ PFU/mL at 1–3 dpi, whereas 3/6 contact ferrets had detectable virus in NW by day 7 postcontact (peak 2.6–4.8 log_10_ PFU/mL; days 9–11 postcontact) (Figure, panel B) as well as viral RNA (6.7–8.2 log_10_ copies/mL), and seroconverted, confirming transmission through the air in 50% of ferrets (3/6). We also detected infectious virus in conjunctival and rectal samples from inoculated animals, but only from 2 contact animals (Table).

**Figure:**
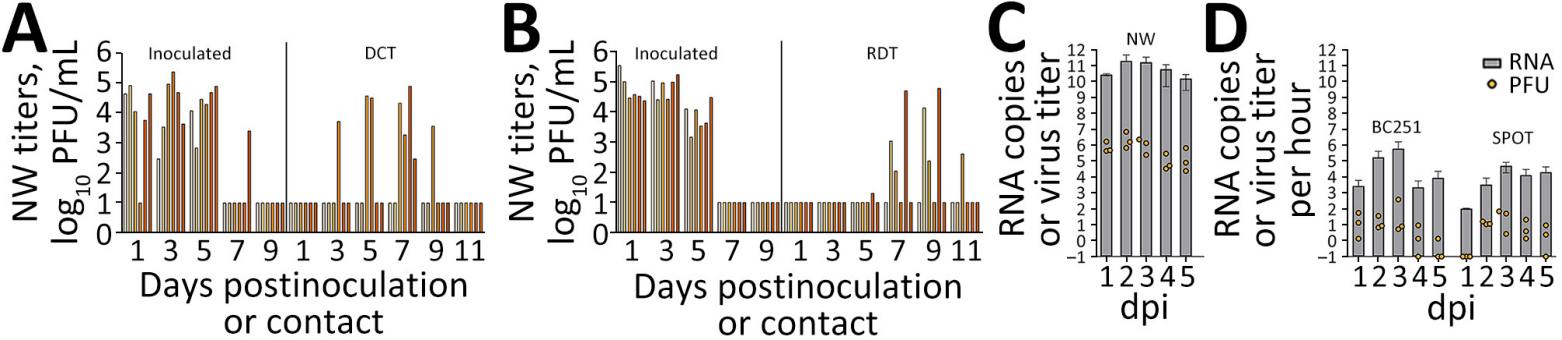
Transmission and measurement of airborne avian influenza A(H5N1) virus isolated from dairy farm worker, Michigan. A, B) For DCT and RDT testing, ferrets (n = 12) were intranasally inoculated with 10^6^ PFU A/Michigan/90/2024 virus, isolated from the dairy worker, in 1 mL phosphate-buffered saline and were cohoused with naive ferrets in a DCT model (A) or in adjacent cages with perforated sidewalls permitting airborne virus spread but restricting contact in an RDT model (B). Each bar represents a single animal. C, D) For aerosol transmission testing, ferrets (n = 3) were inoculated intranasally with 10^6^ PFU of MI90 virus and tested daily (C). Orange dots represent viral titers from NW in log_10_ PFU/mL; limit of detection 10 PFU/mL. Gray bars show average viral M gene RNA load. Error bars indicate SD. Limit of detection was 2.9 log_10_ RNA copies/mL. D) Aerosol samples were collected daily for 5 dpi by using a BC251 cyclone-based sampler (kindly provided by Dr. William Lindsley, National Institute for Occupational Safety and Health) and the SPOT water condensation sampler (Aerosol Devices, https://aerosoldevices.com), as described previously (*8*). Orange dots represent log_10_ PFU/mL per hour. Gray bars show average viral M gene RNA. Error bars indicate SD. Limit of detection was 2.5 log_10_ RNA copies/h. Ferrets were used for tissue collection on day 5. DCT, direct contact transmission; dpi, days postinoculation; NW, nasal washes; RDT, respiratory droplet transmission.

To further evaluate the level of virus exhaled by MI90-inoculated ferrets and the potential for airborne transmission, we collected aerosol samples 1 time each day at 1–5 dpi for 1 hour from the 3 ferrets that were euthanized at 5 dpi. Air samples were analyzed for infectious virus and viral RNA by using the BC251 cyclone-based sampler (kindly provided by Dr. William Lindsley, National Institute for Occupational Safety and Health) and the SPOT water condensation sampler (Aerosol Devices, https://aerosoldevices.com), as described previously (*8*) (Figure, panel D). The highest mean titer of virus was detected at 2 dpi in NW collected from all 3 inoculated ferrets (6.5 log_10_ PFU/mL) (Figure, panel C). Airborne virus was highest at 3 dpi as measured in both samplers, up to 133 and 41 PFU/hour, supporting transmission observed in both contact models within 3–5 days after exposure.

Overall, MI90 virus displayed reduced virulence in ferrets compared with another H5N1 virus isolated from a dairy farm worker in Texas (*8*,*9*); the Texas virus possesses a genetic marker in the polymerase basic 2 protein (E627K), known for enhanced replication and pathogenesis in mammals. At this position, MI90 encodes 627E, like most other viruses isolated from cattle, and contains polymerase basic 2 M631L, which is associated with mammal adaptation (*3*,*9*). In addition, polymerase acidic 142N/E has been linked to increased virulence in mice (*10*); the Texas virus has an E and MI90 virus has a K at this position. Both viruses have identical hemagglutinin sequences associated with receptor binding and the multi-basic cleavage site. Despite differences in virulence, both viruses transmitted in the ferret model with similar proficiency and levels of airborne virus. 

Because avian H5N1 viruses cross the species barrier and adapt to dairy cattle, each associated human infection presents further opportunity for mammal adaption. This potential poses an ongoing threat to public health and requires continual surveillance and risk assessment of emerging viruses to improve our ability to predict and prepare for the next influenza pandemic.
